# GenVarLoader: an accelerated dataloader for applying deep learning to personalized genomics

**DOI:** 10.1186/s12864-026-13225-5

**Published:** 2026-07-24

**Authors:** David Laub, Aaron Ho, Loukik Raina, Jeff Jaureguy, Adam Klie, Rany M. Salem, Graham McVicker, Hannah Carter

**Affiliations:** 1 Bioinformatics and Systems Biology Program, University of California, San Diego, La Jolla, CA 92093 United States; 2 Integrative Biology Laboratory, Salk Institute for Biological Studies, La Jolla, CA 92093 United States; 3 Department of Computer Science and Engineering, University of California, San Diego, La Jolla, CA 92093 United States; 4 Herbert Wertheim School of Public Health and Longevity Science, University of California, San Diego, La Jolla, CA 92093 United States; 5 Department of Medicine, Division of Genomics and Precision Medicine, University of California, San Diego, La Jolla, CA 92093 United States

**Keywords:** Deep learning, Personalized genomics, Variant effect prediction

## Abstract

**Background:**

Deep learning models can predict the functional impact of genetic variants. However, training and applying these sequence models to personalized genome sequences are severely constrained by the computational burden of storing and reading massive datasets.

**Results:**

We developed GenVarLoader, an accelerated dataloader that creates personalized genomic sequences and functional tracks on-the-fly to overcome these bottlenecks. GenVarLoader stores this personalized genomic data in formats specifically optimized for machine learning. Our tool reads variants up to 26,000 times faster than a pre-subset BCF, loads personalized data up to 130 times faster than tuned FASTA and pyBigWig pipelines, and reduces storage approximately 2,000-fold compared to existing alternatives.

**Conclusions:**

By eliminating severe data loading bottlenecks, GenVarLoader ensures efficient model training and inference. It provides a scalable solution for integrating biobank-scale personalized genomic data with deep learning applications.

**Supplementary Information:**

The online version contains supplementary material available at https://doi.org/10.1186/s12864-026-13225-5.

## Background

Deep learning (DL) sequence models trained on reference genome sequences have improved our ability to predict the effects of noncoding variants on gene expression [1], transcription factor binding [2], histone modifications [1], chromatin accessibility [3], and DNA contact maps [4]. However, these models fail to explain individual variation in gene expression [5, 6]. Recent studies address this by training sequence models on personalized genomes paired with gene expression [7–11], leading to more accurate gene regulatory predictions and improved understanding of variant driven disease risk [12]. As they improve, DL sequence models may complement current methods for imputing functional data for phenotypic association studies (e.g. transcriptome-wide association studies) and for predicting phenotypic data directly (e.g. polygenic risk scores). This sets the stage for the application of sequence models to personalized data from biobanks such as UK BioBank [13] and All Of Us (AoU) [14], which contain hundreds of thousands of individuals’ genomic and phenotypic data. However application of DL sequence models to personalized genomes is currently extremely computationally-intensive due to the petabyte-scale of personalized genomic data and slow read throughput.

Efficient methods for generating personalized genomes and functional tracks are essential to scale the training and application of DL sequence models. Most approaches to train on personalized genomes have used bcftools [15] to reconstruct diploid genomes as FASTA files after taking a reference genome FASTA and variant call format (VCF) files as input [7, 8, 16]. Similarly, RNA-seq read depth is typically stored in BigWig format with regions specified in BED files [17]. However, this process demands excessive storage, limits throughput, and lacks support for indels. Writing personal genomes as FASTA files inflates storage requirements by orders of magnitude compared to VCF files. For example, storing the one million genomes from the AoU project would require 1.97 petabytes of storage and cost over $50,000 per month (Supp. Methods). Additionally, FASTA-based data loading limits throughput, lowers GPU utilization, inflates compute costs, and delays model iteration. Finally, supporting indels also requires re-aligning functional tracks and no published tools currently support this.

Here, we introduce GenVarLoader (GVL), which generates personalized genomes and functional tracks on-the-fly, supports indels, and reads and loads data substantially faster than existing approaches. By eliminating data loading bottlenecks, GVL ensures efficient model training and inference. Additionally, GVL is scalable, with sustained performance on datasets of hundreds of.

thousands of genomes.

### Implementation

**Fig. 1:**
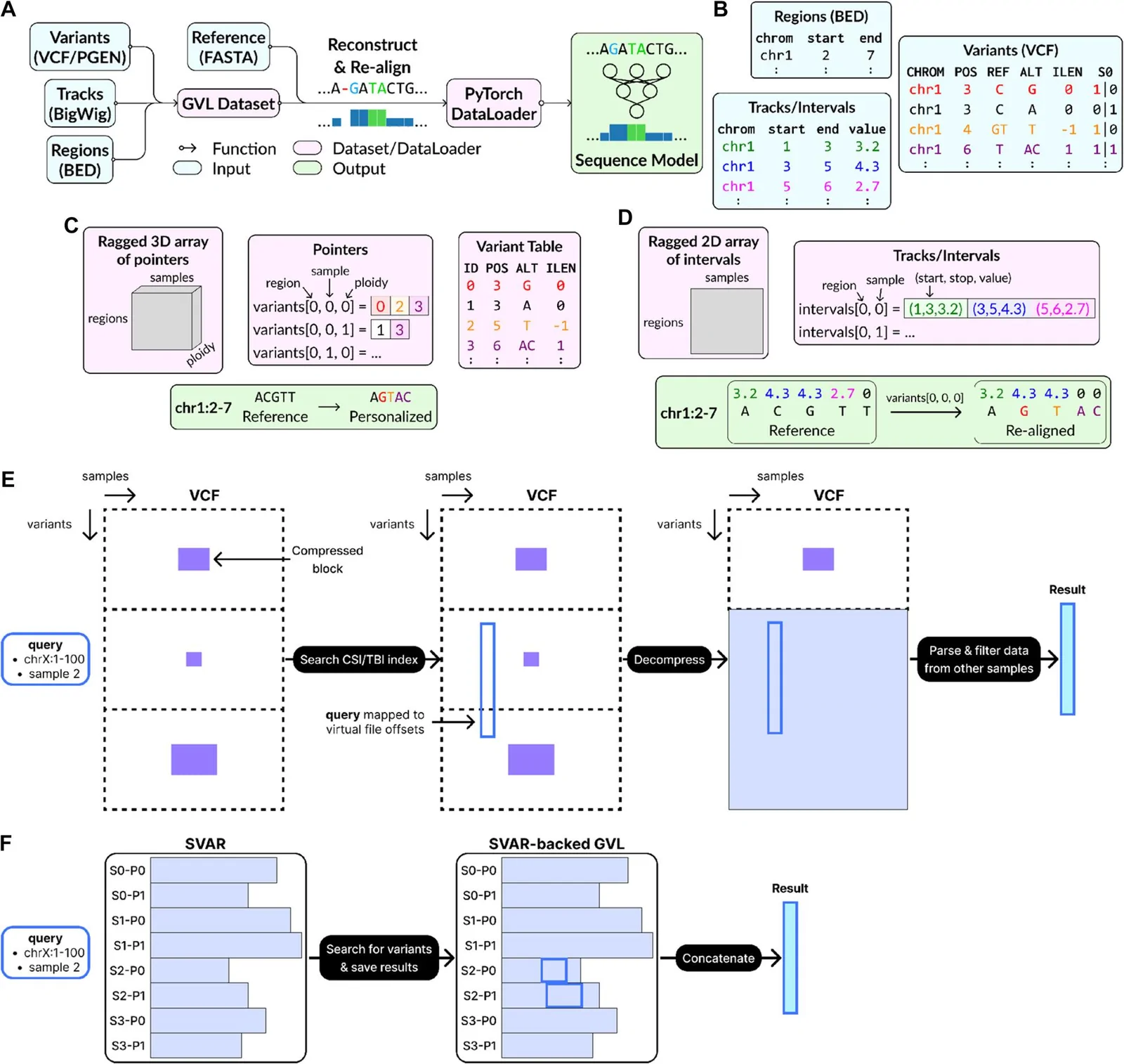
Overview of GenVarLoader data structures and how they compare to standard formats. **A** GenVarLoader (GVL) workflow. Variants, read depth, and regions of interest are converted into a GVL dataset. The GVL dataset and a reference genome are used during training and inference to reconstruct personalized sequences and re-align tracks for sequence models. Tracks are re-aligned to support indels, skipping values overlapping deletions and duplicating values for insertions. **B** Example of source data and how (**C**) variants and (**D**) tracks/intervals are represented in a GVL dataset and used in personalized and re-aligned sequences and tracks. **E** Example of querying data from a VCF with major computational steps highlighted. In contrast, **F** shows how the Sparse Variant (SVAR) format used by GVL eliminates all of these steps, enabling caching search results and zero-copy reads

To easily integrate with common workflows, GVL takes VCF, BigWig, and BED files as input and yields a PyTorch compatible dataloader (Fig. 1A). GVL supports indels by re-aligning the read depth to changes in genome length and adjusting the coordinates for regions of interest. In addition, it reconstructs personalized genomes and re-aligned read depth on-the-fly. GVL returns plain NumPy arrays, so it is framework-agnostic and compatible with frameworks beyond PyTorch.

To optimize throughput, GVL reorganizes variants and BigWig files into uncompressed sample-major arrays. In contrast, VCF and PGEN files store genotypes as dense, variant-major matrices, which are inefficient for randomly sampling regions across individuals (Fig. 1B). Similarly, BigWig files store data per sample, needing expensive operating system calls when a different file is accessed (Fig. 1B). Instead, GVL sparsifies genotypes matrices and rearranges data as uncompressed sample-major arrays (Fig. 1C-D). Typical queries for machine-learning models are variants for a genomic slice for a single sample. We show the steps required to obtain the result from a VCF (Fig. 1E) compared to the Sparse Variant (SVAR) format we created for GVL (Fig. 1F). GVL data formats eliminate the need for search and decompression and use a sample-major layout that optimizes data locality that should result in substantially faster data retrieval.

GVL re-aligns functional tracks to indels using a reference-to-haplotype position mapping similar to prior work [11**]**. Values overlapping a deletion are skipped, and positions created by an insertion are assigned a fill value. The default fill duplicates the value upstream of the insertion. GVL also provides alternative fill strategies for inserted positions: a signal-mass-preserving duplication, a constant value, a value sampled from the flanks, and linear interpolation across the insertion. These strategies let a user control how inserted positions affect downstream models, since duplicating values can inflate signal mass and may create artifacts in convolutional or attention models.

## Results

**Fig. 2:**
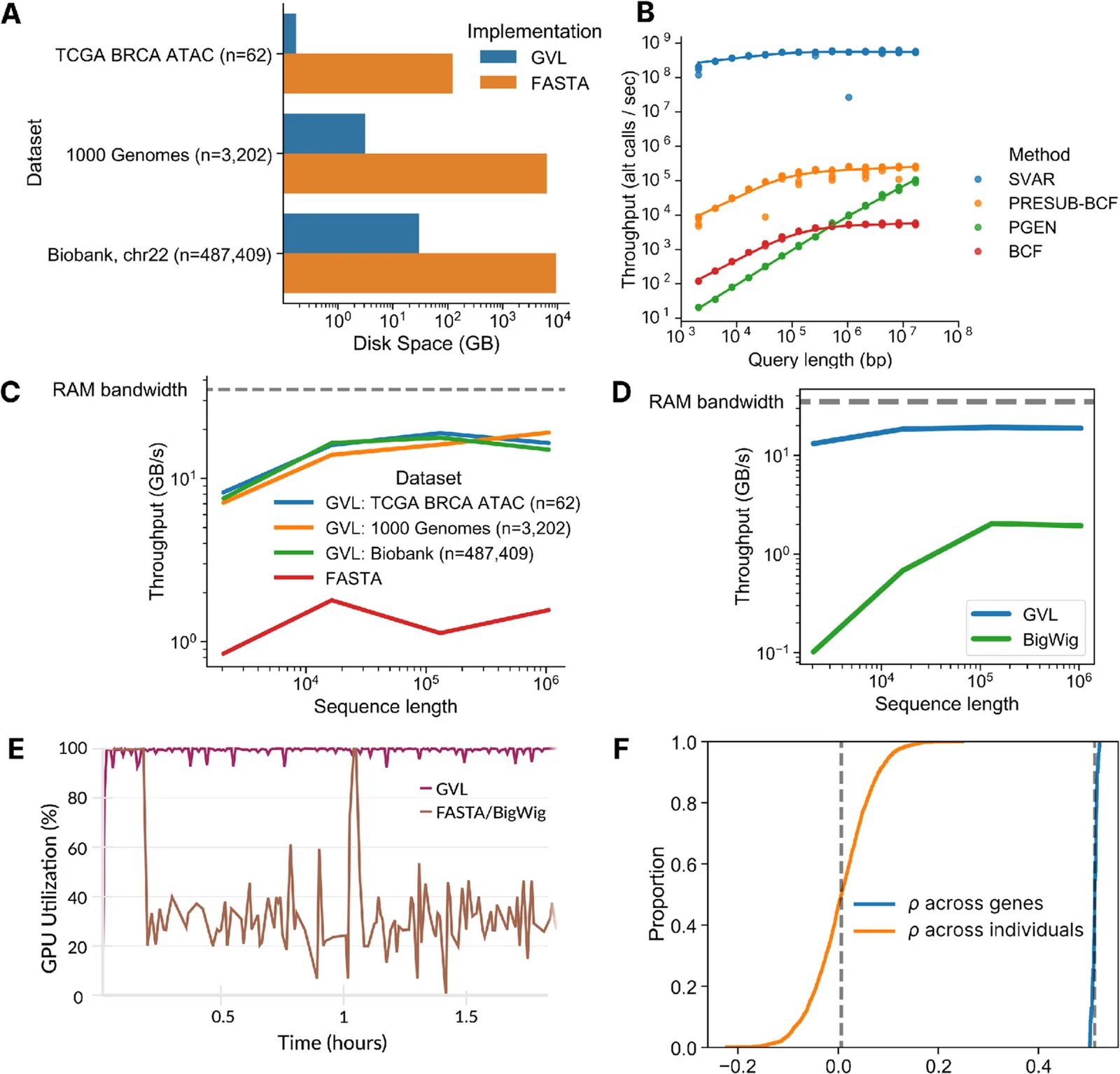
Advantages of GenVarLoader (GVL) over alternatives for storage, throughput, and ease-of-use. **A** Storage requirements for personalized genomes in GVL compared to compressed FASTA files. **B** Time to query a random region and sample for the file type underlying GVL, SVAR, and standard formats, BCF and PGEN, including a pre-subset BCF, across 1,000 Genomes subsets of increasing sample number. **C** GVL throughput for reconstructing haplotypes versus reading FASTA from bcftools; GVL is 7.8 to 16.8 times faster. **D** Throughput for generating re-aligned tracks with GVL versus reading BigWig files; GVL is 9.4 to 128.9 times faster. **E** GPU utilization during training using GVL versus standard tools; GVL enabled full GPU utilization, a 3x improvement. **F** GVL simplifies the evaluation of genomic foundation models. For example, reproducing the evaluation of Basenji2 for personalized gene expression by Huang et al. [5] showing an average Spearman’s ρ of 0 across individuals despite an average ρ close to 0.5 across genes

We benchmarked GVL’s throughput and disk footprint using three datasets of varying size: (1) 62 whole genome sequencing (WGS) and ATAC-seq samples from The Cancer Genome Atlas (TCGA) [18, 19]; (2) 3,202 WGS samples from the 1,000 Genomes Project; (3) 487,409 imputed genotype samples from the UK BioBank limited to chromosome 22. We compared GVL’s storage footprint to storing personalized genomes as two compressed FASTA files, corresponding to prior approaches [7, 8]. For the 1,000 Genomes Project [20], personalized FASTA files required 6.3 TB whereas GVL required only 3.1 GB, an over 2,000-fold reduction, with similar results for the UK BioBank [13] (Fig. 2A). For throughput, we first measured the number of variants per second that could be read from BCF, PGEN, and SVAR files for the 1,000 Genomes Project (Fig. 2B). Because querying a whole-cohort BCF is not a matched comparison, we also queried a BCF whose single-sample, region split was prepared ahead of time. Against this pre-subset BCF, SVAR read variants up to 26,000 times faster for short queries, declining to approximately 1,700 times faster for queries of 1 Mbp and longer. The speedups against unprepared BCF and PGEN files were larger by orders of magnitude, because those formats pay search and decompression costs on every query while SVAR caches them. Variant-read throughput scaled with the number of samples when we subset the 1,000 Genomes Project from 10 to 3,202 individuals. SVAR and the pre-subset BCF maintained cohort-independent read throughput, whereas BCF and PGEN throughput fell roughly 2.4- and 3.9-fold from 10 to 3,202 samples. Peak memory and read throughput for each format as a function of query length and cohort size are detailed in Supp. Figure 4. We then compared the maximum throughput of GVL to reading FASTA files from bcftools using up to 32 threads and 96 GiB of total RAM. The FASTA baseline was a tuned, map-style PyTorch DataLoader with parallel workers, so the comparison reflects a fair baseline. To reflect common lengths used in practice and to assess different data access patterns, we tested sequence lengths ranging from 2 kbp to 1 Mbp. GVL achieved 7.8 to 16.8 times higher throughput than reading FASTA and approached but did not exceed the maximum input bandwidth of GPUs such as the NVIDIA A100 (Fig. 2C). GVL’s throughput was proportional to the amount of data being processed at a time (Supp. Figure 1A). GVL had consistent throughput across all three datasets, demonstrating its scalability to hundreds of thousands of samples or more.

Similarly, we benchmarked GVL’s performance for generating functional track data re-aligned to personal genomes. Using the same resources as before (up to 32 threads and 96 GiB total RAM), we compared GVL against a tuned pyBigWig DataLoader on the TCGA dataset [21]. Testing sequence lengths ranging from 2 kbp to 1 Mbp, we observed 9.4 to 128.9 times faster throughput compared to the pyBigWig implementation (Fig. 2D), again approaching but not exceeding A100 GPU input bandwidth. As with haplotypes, track throughput was proportional to the amount of data processed at a time (Supp. Figure 1B).

We next tested whether GVL could eliminate data loading bottlenecks during model training by achieving full GPU utilization. Using the TCGA ATAC-seq dataset, 32 CPUs, 8 GB total RAM, and one A30 GPU, we compared GVL to a fully optimized data loading pipeline built with pysam [22] and pyBigWig [21] using PyTorch multiprocessing dataloaders. We summed the predictions for the two haplotypes. GVL sustained full GPU utilization while pysam and pyBigWig could only sustain ~ 30% utilization (Fig. 2E).

Finally, to demonstrate GVL’s fidelity and ease-of-use we reproduced the evaluation of Basenji2 by Huang et al. [5] showing that Basenji2 generally fails to predict differences in gene expression across individuals (Fig. 2F). GVL substantially streamlined the process of providing personalized genomic data into Basenji2. In contrast to the pipeline from Huang et al., which was hard coded for regions centered on gene transcription start sites and involved manual handling of multiple external tools (e.g., bcftools and samtools), file-system-based management of intermediate files, and complex indexing strategies, GVL offers a simplified, flexible Pythonic workflow. GVL transparently combines variant selection, personalized genome construction, and data loading into a unified, familiar, and performant interface. This reproduction is included in GVL’s online documentation and demonstrates concise dataset preparation and direct model inference without explicit management of intermediate sequence files or parallelization complexities, making it more user-friendly and reproducible compared to the custom pipelines used in prior work.

We profiled memory use during both dataloading and SVAR conversion. Resident set size during dataloading rose as the memory-mapped genotype and track working set became resident, across four sequence lengths for the 1,000 Genomes and TCGA datasets (Supp. Figure 2). Resident set size can exceed the requested memory without job failure, because most resident memory is reclaimable memory-mapped file data that the operating system evicts on demand. This memory is not swapped to disk. During SVAR conversion, average and peak resident set size were 47 and 86 GiB for the 1,000 Genomes Project from PGEN, and 4 and 6 GiB for TCGA from BCF (Supp. Figure 3).

## Conclusions

GenVarLoader addresses critical barriers in scaling sequence models to personalized genomics. By reducing storage requirements, adding indel support, and substantially improving read and load throughput over existing tools, GVL enables efficient training and inference with DL sequence models on biobank-scale genomic datasets.

GVL applies a post-hoc coordinate transform of functional tracks. It does not re-align reads to a fully reconstructed personalized genome, which would be more accurate. The duplication of values at inserted positions can inflate signal mass and may create artifacts in convolutional or attention models, though the alternative fill strategies described above let users mitigate this effect. Directly validating this post-hoc re-alignment is a natural follow-up: functional data could be aligned to both the reference and a personalized genome assembly, peaks called on the personalized alignment, and GVL’s re-aligned track compared against the personalized-alignment track at a peak overlapping an indel. GVL is also not optimized for CPU-only inference. When no GPU is available, a dataloader should perform close to zero computation so that compute serves the model, and GVL instead targets GPU saturation because the GPU is typically the most expensive hardware in a training run.

Future work will support alternative variant formats (e.g. Hail [23], TileDB-VCF [24]) and variant calls from pangenome and multiple references [25]. Compact variant encodings such as VariantKey [26] could shrink or inline the variant table to reduce RAM. Finally, as GPU hardware evolves, further optimizations can be pursued to ensure GPU bandwidth saturation.

## Availability and requirements

Project name: GenVarLoader.

Project home page: https://pypi.org/project/genvarloader/ and https://genvarloader.readthedocs.io/en/latest/.

Operating system(s): Linux.

Programming language: Python.

Other requirements: PyTorch.

License: MIT license.

Any restrictions to use by non-academics: None.

## Supplementary Information

Supplementary Material 1.

## Data Availability

The datasets analyzed are available in public repositories. TCGA WGS VCFs and ATAC-seq BAMs are available at the Genomic Data Commons portal (https://portal.gdc.cancer.gov/). The 1,000 Genomes Project WGS variant data is available at https://www.internationalgenome.org/data-portal/data-collection/30x-grch38. Imputed UK Biobank data were obtained under project ID 37671 and are available to qualified investigators at https://www.ukbiobank.ac.uk/. GVL datasets for benchmarking the 1,000 Genomes Project are available on Zenodo (https://doi.org/10.5281/zenodo.14367502). Code for benchmarking and results reproduction is archived at Zenodo (https://doi.org/10.5281/zenodo.14375325) and GitHub (https://github.com/d-laub/gvl-paper).

## Abbreviations

AoU: All Of Us
DL: Deep learning
GVL: GenVarLoader
SVAR: Sparse Variant format
TCGA: The Cancer Genome Atlas
VCF: Variant call format
WGS: Whole genome sequencing
