# Supplementary material for: GenVarLoader: an accelerated dataloader for applying deep learning to personalized genomics
Source: BMC Genomics. 2026 Jul 24;27:871. doi: 10.1186/s12864-026-13225-5 (PMC13682130; doi:10.1186/s12864-026-13225-5)
Supplement: Supplementary file 1 [file 12864_2026_13225_MOESM1_ESM.docx]

## Supplementary Methods

### **Benchmarking datasets**

We benchmarked GVL utilizing three datasets encoded as VCF, PGEN, or BigWig files: (1) paired WGS and ATAC-seq data for 62 breast cancer samples from TCGA representing 4,492,345 unique variants^1,2^ ;(2) WGS data for 3,202 individuals from the 1,000 Genomes Project^3^ containing 73,452,337 unique variants; (3) imputed genotypes for 487,409 samples were obtained from the UK BioBank^4^ consisting of 1,247,115 unique variants restricted to chromosome 22.

### **Benchmarking approach**

To compare the storage requirements of GenVarLoader (GVL) to a bcftools consensus-based approach (denoted FASTA), we estimated the size needed by bcftools as the compressed GRCh37 reference genome (987 MB) multiplied by twice the number of individuals for diploid genomes. We measured GVL’s storage using dust^5^ v1.1.1.

We measured the throughput for reading variants from Sparse Variant (SVAR), BCF, and PGEN files using all variants from the 1,000 Genomes Project. All three formats were read in-process through genoray, GenVarLoader’s companion genotype-query library, which exposes a uniform NumPy range-query interface over BCF (via cyvcf2 and HTSlib), PGEN (via pgenlib), and the sparse, memory-mapped SVAR format. Reading every format through the same library makes the comparison reflect the underlying file formats rather than differences in tooling. Random queries, each consisting of a genomic interval and a single sample, were generated. For each query we selected that sample and read its genotypes over the interval (VCF.read for BCF, PGEN.read for PGEN, and a SparseVar read for SVAR), and we report throughput as the number of non-reference allele calls retrieved per second. The genomic intervals ranged in length at powers of two from 2,048 bp to 16,777,216 bp and we timed 5 random queries for each length. For SVAR, we did not include the time for the vectorized index search that locates each query’s variants, since this search is cached in a GVL dataset ahead of training. The timed operation is the read of the genotypes from the memory-mapped store. To provide a matched comparison for this cached behavior, we additionally benchmarked a pre-subset BCF whose single-sample, region split was prepared ahead of time with bcftools view -s <sample> -r <region> --min-ac 1 --no-update, and then read with cyvcf2, so that the timed query did not pay repeated search and decompression costs. A fully equivalent comparison against per-sample, region-split BCFs for arbitrary queries would require software that does not currently exist. To measure how throughput scales with cohort size, we repeated the SVAR, BCF, PGEN, and pre-subset BCF benchmarks on random subsets of the 1,000 Genomes Project of 10, 32, 100, 316, 1,000, and 3,202 individuals.

To measure personalized sequence throughput, we ran benchmarks on a SLURM-based high-performance computing cluster (node cn-03) allocating 96 GiB of total RAM and 1, 4, 16, or 32 threads from an AMD EPYC 7543 32-Core processor. We selected genome-wide non-overlapping intervals at sequence lengths of 2,048, 16,384, 131,072, and 1,048,576 nucleotides. Batch sizes were chosen at powers of two ensuring that batches did not exceed 8 GB. Each benchmarking run proceeded until 2^29 base pairs were processed or 10 batches were completed, whichever was larger, and each configuration was repeated 5 times. The maximum throughput measured across threads, batch sizes, and replicates is shown in Supplementary Figure 1A and 1B. The FASTA and BigWig baselines were implemented as tuned, map-style PyTorch DataLoaders with the number of worker processes set to 31 of 32 cores and a full batch-size sweep, on the same node, region grid, and timing loop as GVL. We read FASTA with pysam v0.22.1 and BigWig with pyBigWig v0.3.23. The FASTA baseline reads an uncompressed FASTA, assuming bcftools consensus was run ahead of time, and reads two haplotype files per locus to reproduce GVL’s diploid output. It evicts the local page cache after each read, so its throughput is an upper bound and the reported speedup a lower bound.

To assess GPU utilization, we trained a convolutional neural network on one-hot encoded DNA sequences of 2,048 bp to predict 1,048 bp of ATAC-seq read depth centered at peaks. The model architecture is adapted from BPNet^6^ using an initial convolutional layer with a kernel size of 21, followed by 8 dilated convolutional layers. Predictions for each haplotype were summed together to make the final prediction, following the practice of combining per-haplotype predictions in models trained on personalized genomes^7,8^. Each training run was allocated 32 CPUs, 8 GB RAM, and one NVIDIA A30 GPU. GPU utilization was measured with Weights & Biases.

### **SVAR conversion time**

We measured the wall-clock time to convert each dataset into the SVAR format used by GVL. Converting the 1,000 Genomes Project from PGEN took approximately 74 minutes using 8 parallel jobs, and converting TCGA from BCF took approximately 178 seconds. The throughput measurements in the main text exclude this one-time preprocessing step.

### **Validating reconstructed haplotypes**

We validated GVL’s reconstructed haplotypes against bcftools consensus. We compared GVL output to bcftools consensus -H 2 on real 1,000 Genomes data and on a curated synthetic set. We additionally use property-based testing with the Hypothesis library [CITATION] against randomly generated, reference-consistent VCF inputs.

### **Estimating cloud storage costs**

We estimated cloud storage costs for 1 million personal genomes stored as compressed FASTA files using the AoU pricing of $0.026 per GB⋅month and the GRCh37 reference genome size (987 MB). Each individual requires one FASTA file per ploidy, totaling 1,000,000 individuals x 0.987 GB/ploidy x 2 ploidy/individual x $0.026/GB⋅month = $51,324/month.

### **Evaluating Basenji2 on personal gene expression**

To reproduce results from Huang et al.^9^, we obtained evaluation data from the Geuvadis consortium via EMBL-EBI BioStudies accession E-GEUV-1. The data includes paired WGS genotypes and RNA-seq gene expression from 465 individuals. All steps exactly match Huang et al. except for averaging predictions from the forward and reverse complements as well as shifts of 1 nucleotide to the left and right. Instead, doing a single forward pass matching each gene’s biological orientation was more computationally efficient and sufficient to match their results.

## Figures

Supplementary Figure 1. GenVarLoader throughput is roughly proportional to the amount of data processed at a time and the number of threads used. A) Throughput for generating personalized DNA sequences. GVL has higher throughput than reading FASTA files once ~2,000,000 nucleotides are being generated at a time. B) Throughput for generating personalized tracks.

**Supplementary Figure 2.** Memory usage during dataloading as a function of sequence length, dataset, and output type.

Each panel shows one requested output sequence length (2, 16, 128, or 1,024 kb). Within each panel, each line is one data-loading run on a single node (32 threads, 96 GiB memory requested, shown by the red dotted line labeled “Alloc. RAM” in the legend). Color indicates the dataset: 1000 Genomes Project genotypes (1kGP, blue) and TCGA-ATAC (orange). Line style indicates the output type: personalized haplotype sequences (solid) or base-pair-resolution coverage tracks (dashed). 1kGP provides only haplotypes. TCGA-ATAC provides both. All runs used the eager loader (mode=none), in which every mini-batch is decoded from disk on demand, so the curves reflect the memory cost of producing batches rather than of reusing pre-decoded data. Batch size was scaled inversely with sequence length to hold the number of base pairs per batch approximately constant.

Axes. The y-axis is resident set size (RSS), the physical RAM held by the loader process, in GiB. The x-axis is the cumulative number of instances decoded (millions), where one instance is a (region, sample) pair. We plot RSS against instances decoded rather than wall-clock time because all runs were given the same 180 s budget, and the number of instances produced within that budget differs by orders of magnitude across sequence lengths (a 1,024 kb instance contains 512 times more sequence than a 2 kb instance and takes correspondingly longer to decode). Plotting against instances decoded normalizes for this difference and shows how far each run progressed as the horizontal extent of its curve.

Memory model. GVL does not materialize whole personalized genomes. The reference genome is loaded once into RAM (approximately 3 GiB for GRCh38) and contributes a fixed baseline that is independent of sequence length. The genotype store and the coverage-track intervals are instead memory-mapped: the operating system exposes each file as addressable memory but loads a given region into RAM only when it is first accessed, and may evict it again later. Two quantities therefore determine RSS: (i) the working set, the amount of memory-mapped genotype or track data that has been accessed and remains resident, and (ii) the decoded batch, the dense output array assembled for the current mini-batch, whose size equals (instances per batch) × (sequence length) × (bytes per base pair) and which is allocated and freed on each iteration. Different datasets and output types load these two quantities differently, which accounts for the qualitatively different curves.

1kGP haplotypes are working-set-limited. 1kGP contains approximately 7.7 million instances backed by a large genotype store, and RSS is dominated by quantity (i). At 2 to 128 kb, decoding is fast enough that the loader traverses the full dataset one or more times within 180 s, the genotype store becomes fully resident, and RSS plateaus near 115 GiB. At 1,024 kb, each instance is more expensive to decode, the loader completes only about 13% of a single pass (note the shorter blue curve), a correspondingly smaller fraction of the genotype store is accessed, and peak RSS is lower (approximately 54 GiB). The largest sequence length therefore uses less memory because the fixed time budget accesses less of the data, not because long sequences are intrinsically cheaper. The UKBB dataset, which is also genotype-only and produces haplotypes but contains far more instances, occupies this same working-set-limited regime and is omitted from the figure as redundant. It would differ from 1kGP only in the larger absolute size of its genotype store and the correspondingly smaller fraction of it accessed within the time budget.

TCGA-ATAC is batch-size-limited. TCGA-ATAC contains approximately 147,000 instances, so the loader completes many full passes at every sequence length and coverage is never limiting. RSS is instead dominated by quantity (ii). At short sequence lengths the batch size is capped by the dataset size (a batch cannot exceed approximately 147,000 instances), so the decoded batch grows in proportion to sequence length and peak RSS rises accordingly (haplotypes: 6 GiB at 2 kb, 13 GiB at 16 kb). Once the sequence length is large enough that the configured batch size falls below the dataset size, holding base pairs per batch constant fixes the decoded batch size, and peak RSS for haplotypes saturates (approximately 36 GiB at both 128 and 1,024 kb). Coverage tracks reach higher RSS than haplotypes for two reasons: each track base pair is stored as a 4-byte value versus approximately 2 bytes for a diploid haplotype base, and the resident track working set continues to grow with sequence length because longer windows access more of the genome-wide track data (approximately 74 GiB at 128 kb, 111 GiB at 1,024 kb), whereas the sparse genotype working set saturates.

Additional features. The rapid oscillation on each plateau reflects the repeated allocation and release of the decoded batch on each iteration, not disk swapping. Peak RSS exceeds the 96 GiB request in several configurations (1kGP haplotypes at 2 to 128 kb, and TCGA-ATAC tracks at 1,024 kb). This is possible without job failure because most of the resident memory in these cases is memory-mapped file data, which the operating system can reclaim on demand. The decoded batch, in contrast, is non-reclaimable. RSS drops to approximately 4 GiB at the end of every run once the loop terminates and the final batch is released, returning the process to its baseline footprint.

**Supplementary Figure 3.** Memory usage during conversion of source data into the SVAR format. Bars show the average and peak resident set size (RSS) of the conversion process, in GiB, for each dataset. Converting the 1,000 Genomes Project from PGEN used 47 GiB average and 86 GiB peak RSS, and converting TCGA-ATAC from BCF used 4 GiB average and 6 GiB peak RSS.

**Supplementary Figure 4.** Memory and read throughput of single-sample, single-region variant queries scale differently across storage formats. We drew random (region, sample) queries from the 1,000 Genomes Project (3,202 individuals) and retrieved the genotypes from four formats: Sparse Variant (SVAR, the format GVL reads), BCF, PGEN, and a pre-subset BCF (PRESUB-BCF) whose single-sample, single-region split is prepared ahead of time. Each batch held the number of queried base pairs approximately constant, so the number of queries per batch scaled inversely with query length, from 65,536 queries per replicate at 2 kb to 8 at 16 Mb. Each run used 8 CPUs and 64 GB of RAM on one node. Color denotes the format and is shared across all panels. Peak RSS and throughput (non-reference allele calls retrieved per second) are plotted on log–log axes with LOWESS fits.

a) Peak RSS versus query length at full cohort. SVAR peak RSS falls from approximately 89 GiB at 2 kb queries to approximately 10 GiB above 1 Mb (**Supp. Fig. 4a**). SVAR stores genotypes as a single memory-mapped sparse array, so resident memory is the set of file pages the read phase pages in, which grows with the number of distinct queries touched rather than with their length. Holding base pairs per batch constant makes the query count scale as one over query length, so short queries scatter tens of thousands of random accesses across the store and make a large fraction of it resident, while long queries touch little of it. Above roughly 1 Mb the query count bottoms out at tens of queries per batch and SVAR RSS saturates at its memory-map and runtime baseline of approximately 10 GiB. PGEN is flat at approximately 20 GiB because its memory is dominated by the genotype index loaded once at open, after which pgenlib reads by file offset with no growing map. BCF is flat below 0.6 GiB because it streams one record at a time through cyvcf2 and frees each, leaving only a fixed library and buffer footprint. PRESUB-BCF stays below 0.3 GiB and drifts down slightly with query length because each batch processes fewer prepared single-sample files as the query count falls, and each file holds only one sample’s calls in one region.
b) Setup throughput versus query length at full cohort, for the two formats with a distinct setup phase. SVAR setup throughput is flat near 15,000–25,000 calls per second up to approximately 100 kb, then rises steeply to approximately 680,000 calls per second at 16 Mb (**Supp. Fig. 4b**). SVAR setup is the vectorized index search that locates offset ranges, whose cost is dominated by a fixed per-query component. At short query lengths the batch contains tens of thousands of queries and a near-constant number of calls is divided by a search time that tracks this large query count, holding throughput flat. As query length grows the query count collapses to tens, the fixed per-query cost vanishes, and each search resolves a long, call-rich range, so throughput climbs. PRESUB-BCF setup throughput rises steeply from approximately 60 calls per second at 2 kb to approximately 10,000 at 1 Mb, then increases much more slowly to approximately 13,000 at 16 Mb. PRESUB-BCF setup launches one bcftools subprocess per query, so at short query lengths the thousands of process spawns dominate and few calls are returned per spawn. As query length grows fewer subprocesses each return more calls, amortizing the spawn cost, until the per-subprocess index seek and decompression of bcftools becomes the floor and throughput plateaus.
c) Peak RSS versus cohort size at a fixed 131 kb query length. SVAR peak RSS grows roughly tenfold across the cohort, from approximately 1.5 GiB at 10 samples to approximately 16 GiB at 3,202 samples, while the other formats are comparatively flat (**Supp. Fig. 4c**). The SVAR sparse store keeps one entry per (variant, sample) call, so a fixed region pages in genotypes for proportionally more samples as the cohort grows and its working set scales with cohort size. BCF and PRESUB-BCF read a single sample at a time and are independent of cohort size below 0.5 GiB. PGEN rises modestly because its index covers all samples, but its read footprint is otherwise cohort-independent.
d) Read throughput versus cohort size at a fixed 131 kb query length. SVAR and PRESUB-BCF throughput are constant across the cohort while BCF and PGEN throughput fall as cohort size grows, by approximately 2.4-fold and 3.9-fold respectively from 10 to 3,202 samples (**Supp. Fig. 4d**). BCF and PGEN store genotypes for the whole cohort at each site, so a single-sample query must decode a record block whose size scales with cohort size and then discard all but one sample, making per-query work grow with the cohort. SVAR addresses each sample’s calls directly through its sparse offsets, and PRESUB-BCF already paid the cohort-wide scan once during setup (panel b), so both read only single-sample data and their throughput is cohort-independent.

## Supplementary References

1.	Aaltonen, L. A. *et al.* Pan-cancer analysis of whole genomes. *Nature* **578**, 82–93 (2020).

2.	Corces, M. R. *et al.* The chromatin accessibility landscape of primary human cancers. *Science* **362**, eaav1898 (2018).

3.	Auton, A. *et al.* A global reference for human genetic variation. *Nature* **526**, 68–74 (2015).

4.	Sudlow, C. *et al.* UK Biobank: An Open Access Resource for Identifying the Causes of a Wide Range of Complex Diseases of Middle and Old Age. *PLOS Med.* **12**, e1001779 (2015).

5.	andy.boot. bootandy/dust. (2024).

6.	Avsec, Ž. *et al.* Base-resolution models of transcription-factor binding reveal soft motif syntax. *Nat. Genet.* **53**, 354–366 (2021).

7.	Rastogi, R., Reddy, A. J., Chung, R. & Ioannidis, N. M. Fine-tuning sequence-to-expression models on personal genome and transcriptome data. *Genome Biol.* https://doi.org/10.1186/s13059-026-04091-1 (2026) doi:10.1186/s13059-026-04091-1.

8.	Celaj, A. *et al.* An RNA foundation model enables discovery of disease mechanisms and candidate therapeutics. 2023.09.20.558508 Preprint at https://doi.org/10.1101/2023.09.20.558508 (2023).

9.	Huang, C. *et al.* Personal transcriptome variation is poorly explained by current genomic deep learning models. *Nat. Genet.* **55**, 2056–2059 (2023).
